# Is There a Role for Post-Mastectomy Radiotherapy for T1-2N1 Breast Cancers With Node-Positive Pathology After Patients Become Node-Negative Pathology Following Neoadjuvant Chemotherapy?

**DOI:** 10.3389/fonc.2020.00892

**Published:** 2020-06-30

**Authors:** Qian Wang, Jingjing Zhao, Xiaowei Han, Puchun Er, Xiangying Meng, Jinyan Shi, Huiru Sun, Jingyang Zhu, Li Zhu, Shikai Wu, Wencheng Zhang, Bing Sun

**Affiliations:** ^1^Department of Radiation Oncology, The Fifth Medical Center, Chinese PLA General Hospital, Beijing, China; ^2^Department of Radiation Oncology, National Clinical Research Center of Cancer, Key Laboratory of Cancer Prevention and Therapy, Tianjin Medical University Cancer Institute and Hospital, Tianjin, China; ^3^Department of Breast Surgeons, The Fifth Medical Center, Chinese PLA General Hospital, Beijing, China; ^4^Department of Oncology, Peking University First Hospital, Beijing, China

**Keywords:** breast cancer, neoadjuvant chemotherapy, surgery, post-mastectomy radiotherapy, complete pathological response

## Abstract

**Purpose:** To assess the benefit of post-mastectomy radiotherapy (PMRT) in breast cancer (BC) patients with T1-2N1M0 who developed pathologically negative lymph nodes (ypN0) after undergoing neoadjuvant chemotherapy (NAC) and mastectomy.

**Patients and Materials:** Patients with T1-2 tumors and positive lymph node(s) who became pN0 after NAC and mastectomy were screened from our prospectively maintained database. The primary endpoint was recurrence-free survival (RFS), and the secondary endpoints were local recurrence-free survival (LRFS) and overall survival (OS). Propensity-score matching (PSM) was conducted for the comparison between PMRT and non-PMRT groups.

**Results:** Of the 142 eligible patients, 110 (77.5%) received PMRT, and 32 (22.5%) did not. The median follow-up time was 72 months. Univariate analyses showed that the 5-year RFS, LRFS, and OS rates were 88.7, 94.5, and 96.1, respectively, with PMRT and 72.4, 90.1, and 95.0% without PMRT (*p* = 0.028; *p* = 0.151; *p* = 0.971). Multivariate analyses established PMRT as a significant prognostic factor for RFS rate (HR, 0.411; 95% CI, 0.175–0.968; *p* = 0.042). After a PSM analysis (64 in the PMRT group vs. 32 in the non-PMRT group), PMRT remained significant, with improved RFS in univariate and multivariate analysis (with 5-year RFS rates of 90.1 vs. 72.4%, respectively, *p* = 0.016; HR, 0.323, 95%CI, 0.115–0.913, *p* = 0.033). In the subgroup of 48 (33.8%) patients with pathologic complete responses (pCR, ypT0, and ypN0) after NAC, PMRT did not affect RFS (HR, 0.226; 95% CI, 0.034–1.500; *p* = 0.123).

**Conclusions:** PMRT might benefit pT1-2N1M0 patients with pN0 after NAC. Patients with pCR might consider omitting PMRT. Prospective studies are needed to assess the effect of PMRT on this specific patient population.

## Introduction

Neoadjuvant chemotherapy (NAC) is a standard of care for patients with locally advanced or inflammatory breast cancer and is increasingly used to treat patients who have an early stage of the disease (1, 2). The potential efficiency of down-staging challenges the standard indications for post-mastectomy adjuvant radiotherapy (3, 4). It is unclear whether initial clinical stage or residual disease after NAC is the more important factor in predicting locoregional recurrence (LRR). According to findings from previous randomized trials, the use of post-mastectomy radiation therapy (PMRT) can improve the outcomes of selected patients who receive mastectomy in the adjuvant setting (5–7). However, none of the prospective phase III trials conducted to date has investigated the effect of PMRT in patients who received neoadjuvant treatment. Therefore, selecting patients to undergo PMRT after NAC treatment is still contentious and has only followed directions suggested by retrospective analyses (8–13).

Previous studies that have considered the use of PMRT in the NAC setting included breast cancer patients who were clinically node-positive and had stage II–III disease; these patients represent a heterogeneous group with respect to their clinicopathological and treatment response-related characteristics. In 2008, the National Cancer Institute (NCI) released a statement recommending the strong consideration of PMRT in the treatment of clinical stage III breast cancer patients and patients who developed positive nodes confirmed by histology following NAC treatment (14). According to the panel, it was difficult to tell whether clinical stage II breast cancer patients with negative lymph nodes could benefit from PMRT. Recently, the results of studies performed by Liu et al. (12) showed that patients with clinical stage IIIB/IIIC breast cancer, patients with T3/T4 tumors, and those with residual invasive breast cancer benefited significantly from PMRT following an initial treatment with NAC; they had improved OS (*p* < 0.05). A survey of 372 radiation oncologists produced a split decision in which 49.9% recommended PMRT for clinical-stage T2N1 patients who attain pathologically negative nodes (ypN0) after NAC (15).

Given the uncertain treatment indication and the lack of adequate data, we analyzed a large cohort of breast cancer patients to assess the efficacy of PMRT in terms of recurrence-free survival (RFS), local recurrence-free survival (LRFS), and overall survival (OS) in pathological node-positive, stage II (T1-2N1M0) breast cancer patients with ypN0 after NAC and mastectomy.

## Methods and Materials

### Patients

Stage II (T1-2N1M0) breast cancer patients who were diagnosed between January 2004 and December 2016 and who achieved pN0 after neoadjuvant chemotherapy were enrolled in this study. The inclusion criteria were women 18 years or older, histologically confirmed T1-2 (tumor size ≤ 5 cm), pathologically and/or clinically node-positive, and pathologically confirmed complete nodal response at surgery after receiving treatment with NAC. Patients with distant metastasis at diagnosis, clinically positive supraclavicular, or internal mammary lymph nodes, inflammatory or bilateral breast cancer, or other personal histories of other malignancies were excluded. Clinical staging was performed according to the 8th edition cancer staging form for TNM staging developed by the American Joint Committee on Cancer. Patients who were staged according to the 7th edition were restaged to fit the 8th edition. Clinical T stage was determined by imaging (breast ultrasonography and/or magnetic resonance imaging). N stage was confirmed by ultrasound-guided fine-needle aspiration, sentinel lymph node biopsy or imaging (ultrasonography: grade 5A and/or Magnetic resonance imaging) prior to the initiation of treatment. Patients who had integrated clinical information and follow-up were eligible for analyses. Selection based on these criteria resulted in a cohort of 142 patients with the T1-2N1M0 disease and ypN0 following NAC; 110 of these patients received PMRT, and 32 did not. Pathological complete remission (pCR) was defined as the absence of invasive carcinoma or ductal carcinoma *in situ* in the breast (ypT0) or axillary lymph nodes (ypN0) (both negative) of a patient. Our institutional review board approved the review of the patients' medical records for this study.

### Radiotherapy

All radiation treatments were conducted at our center. For patients receiving mastectomy, radiation was delivered to the chest wall and supraclavicular lymph nodes at a total radiation dose of 50–60 Gy. PMRT typically uses a photon field to treat supraclavicular fossa and an electron field to treat the chest wall. The standard RT schedule consisted of daily fractions of 1.8–2.0 Gy.

### Statistical Analysis

Recurrence-free survival (RFS) was defined as the interval from diagnosis to the first disease recurrence in the ipsilateral chest wall or the ipsilateral draining regional lymph nodes (axillary, supraclavicular, infraclavicular, or internal mammary lymph nodes), or any distant metastases (all recurrences at other sites). Local recurrence-free survival (LRFS) was defined as the interval from diagnosis to the first disease recurrence in the ipsilateral chest wall or the ipsilateral draining regional lymph nodes. Overall survival (OS) was defined as the interval from diagnosis to death or to the last follow-up. Matching variables included surgery, biopsy of axillary nodes before NAC, response to NAC, adjuvant chemotherapy, and making the baseline data of subgroups comparable. PSM was performed using the MatchIt package with the nearest neighbors method in R, version 3.5.1 (http://www.r-project.org/). The curves for RFS and OS were constructed using the Kaplan-Meier method and compared using the log-rank test. A Cox regression model was used to perform multivariate analyses that included significant prognostic factors in univariate analyses (*p* < 0.05). The characteristics of the PMRT and non-PMRT groups were compared using the χ^2^ test, Fisher's exact test, or Student's *t*-test. A two-sided *p* < 0.05 was considered statistically significant. The statistical analyses were conducted using the SPSS version 20.0 software (SPSS Inc., Chicago, IL, USA).

## Results

### Patient Characteristics and Treatment

Table 1 summarizes the demographic, clinicopathological, and treatment characteristics of the two groups of the unmatched and matched patient populations. Of the 142 patients with ypN0 status after NAC and mastectomy, 110 (77.5%) received PMRT, and 32 (22.5%) did not. The median age of the entire participating population was 49 years (range 23–66 years), and the median follow-up time was 72 months (range 66–78 months). All patients underwent ultrasonography of the breast and regional lymph nodes prior to chemotherapy. Thirty-five (24.6%) patients were subjected to magnetic resonance imaging. One hundred and sixteen patients (81.7%) had suspicious positive axillary lymph nodes confirmed by ultrasound-guided fine-needle aspiration or sentinel lymph node biopsy; the remaining patients were examined clinically using imaging (ultrasound: grade 5A and/or MRI). More patients in the irradiated group than in the non-irradiated group had the initial N-stage confirmed histologically (92.7 vs. 43.7%, *p* < 0.001). Forty-eight (33.8%) patients achieved pCR after NAC.

**Table 1:** Patient characteristics and treatment.

**Variables**	**All patients**			**After propensity matching**		
	**PMRT group (*n* = 110) no. (%)**	**Non-PMRT group (*n* = 32) no. (%)**	***p*-value**	**PMRT group (*n* = 64) no. (%)**	**Non-PMRT group (*n* = 32) no. (%)**	***p*-value**
Age (y)						
Median(Range)	49 (23–66)	49 (28–62)	0.806	50 (23–62)	49 (28–64)	0.627
Age group (y)						
<50	60 (54.5)	18 (56.3)	0.865	32 (50.0)	18 (56.3)	0.563
≥50	50 (45.5)	14 (43.7)		32 (50.0)	14 (43.8)	
Clinical stage (AJCC)						
IIa	24 (21.8)	8 (25.0)	0.705	17 (26.6)	8 (25.0)	0.869
IIb	86 (78.2)	24 (75.0)		47 (73.4)	24 (75.0)	
Biopsy of axillary nodes before NAC						
Yes	102 (92.7)	14 (43.7)	0.000	56 (87.5)	14 (43.8)	0.000
No	8 (7.3)	18 (56.3)		8 (12.5)	18 (56.3)	
Primary tumor response to NAC (pCR)						
Yes	38 (34.5)	10 (31.3)	0.729	30 (46.9)	10 (31.3)	0.143
No	72 (65.5)	22 (68.8)		34 (53.1)	22 (68.7)	
Median numbers of sampled LN (range)	23 (6–69)	19 (10–48)	0.109	23 (7–69)	19 (10–48)	0.102
Tumor HR status						
Positive	64 (58.2)	17 (53.1)	0.257	35 (54.7)	17 (53.1)	0.885
Negative	46 (41.8)	15 (46.9)		29 (45.3)	15 (46.9)	
Tumor HER-2 status^*^						
Positive	56 (50.9)	10 (31.2)	0.089	32 (50.0)	10 (31.3)	0.106
Negative	48 (43.6)	18 (56.3)		27 (42.2)	18 (56.3)	
Molecular subtype^¶^						
Luminal	64 (58.2)	17 (53.1)	0.985	35 (54.7)	17 (53.1)	1.000
HER-2 overexpressing	21 (19.1)	6 (18.8)		13 (20.3)	6 (18.8)	
Triple negative	21 (19.1)	6 (18.8)		13 (20.3)	6 (18.8)	
LVSI^§^						
Yes	7 (6.4)	1 (3.1)	0.505	5 (7.8)	1 (3.1)	0.680
No	103 (93.6)	30 (93.8)		59 (92.2)	30 (93.8)	
Cycles of NAC						
Median (Range)	6 (2–10)	4 (1–11)	0.850	6 (2–10)	4 (1–11)	0.660
NAC regimen						
Anthracycline-based	2 (1.8)	1 (3.1)	0.637	2 (3.1)	1 (3.1)	0.678
Taxane + anthracycline	90 (81.8)	27 (84.4)		48 (75.0)	27 (84.4)	
Taxane-based	18 (16.4)	4 (12.5)		14 (21.9)	4 (12.5)	
Adjuvant chemotherapy						
Yes	29 (26.4)	21 (65.6)	0.000	29 (45.3)	21 (65.6)	0.06
No	81 (73.6)	11 (34.4)		35 (54.7)	11 (34.4)	
Adjuvant hormonal treatment						
Yes	58 (52.7)	16 (50.0)	0.786	33 (51.6)	16 (50.0)	0.885
No	52 (47.3)	16 (50.0)		31 (48.4)	16 (50.0)	
Adjuvant targeted therapy						
Yes	32 (29.1)	5 (15.6)	0.127	19 (29.7)	5 (15.6)	0.134
No	78 (70.9)	27 (84.4)		45 (70.3)	27 (84.4)	

*AJCC, American Joint Committee on Cancer (2009); HER 2, human epidermal growth factor receptor 2; LN, lymph node; LVSI, lymphovascular space invasion; NAC, neoadjuvant chemotherapy; PMRT, post-mastectomy radiation therapy*.

* *Represents 11 patients with Her-2 (++) whose HER-2 status was not analyzed using fluorescence in situ hybridization (FISH) methods; of these patients, six were in the PMRT group, and four were in the non-PMRT group*.

¶ *Represents four patients whose molecular subtype is unknown in the PMRT group and three patients with unknown molecular subtype in the non-PMRT group*.

§ *Represents the one patient whose LVSI was not established pathologically in the non-PMRT group*.

The most common NAC regimen administered to patients was a combination of anthracycline and taxane (*n* = 117, 82.4%). Thirty patients (21.1%) received anti-human epidermal growth factor receptor-2 (HER-2)-targeted agents in the NAC regimen. Additionally, all patients underwent radical mastectomy and axillary lymph nodes dissection (ALND). The subtype of each patient's surgery was based on multidisciplinary assessments and on the preferences of the patient. More patients in the non-irradiated group than in the irradiated group received adjuvant chemotherapy (65.6 vs. 26.4%, *p* < 0.001). Comparison of age, clinical stage, response to NAC, HR status, HER2 status, LVSI, NAC type, and adjuvant hormonal treatment and targeted therapy between the two groups found no dissimilarities.

After matching with PSM, of the 96 patients with a pN0 status after NAC and mastectomy, 64 (66.7%) received PMRT, and 32 (33.3%) did not. The differences between the PMRT and non-PMRT groups were well-balanced. Apart from the significant difference in the biopsy of axillary lymph nodes established after PSM, there were no other significant differences between the groups of matched patient populations (Table 1). More nodes were confirmed pathologically in the patients in the PMRT groups than in the non-PMRT groups (87.5 vs. 43.8%, *p* < 0.001).

### Patterns of Failure

At the time of the last investigation stage for the present analysis (May 2019), 8 patients (5.6%) had died [6 (5.5%) in the PMRT group and 2 (6.3%) in the non-PMRT group; *p* = 1.000], and 22 patients (15.5%) had relapsed (locoregional recurrence in 9, distant metastases in 12, concurrent locoregional recurrence and distant metastasis in 1). Of the 22 patients who relapsed, 10 patients (7.0%) had developed LRR (locoregional recurrence): six in the PMRT group and four in the non-PMRT-group. Thirteen (9.2%) patients were diagnosed with distant metastasis: seven in the PMRT group and six in the non-PMRT group. The details of the pattern of relapse are presented in Table 2. There were no statistically significant differences in the patterns of failure between the two groups.

**Table 2:** Patterns of failure.

**Initial recurrent sites**	**PMRT** **(*n* = 110)**	**Non-PMRT** **(*n* = 32)**	***p*-value**
Locoregional	6^*^ (5.5%)	4^†^ (12.5%)	0.328
Chest wall	3	2	
Supraclavicular LN	4	1	
Axillary LN	2	1	
Internal mammary LN	0	1	
Distant metastasis	7 (6.4%)	6 (18.8%)	0.073

*LN, lymph node; PMRT, post-mastectomy radiation therapy*.

*Represent the one patient who had supraclavicular, internal mammary LN recurrence and distant metastasis*.

* *Represents the one patient who had chest wall, supraclavicular, and axillary LN recurrence. Another patient had chest wall and supraclavicular LN recurrence*.

### Univariate and Multivariate Analyses of the Entire Population

**Figure 1:**
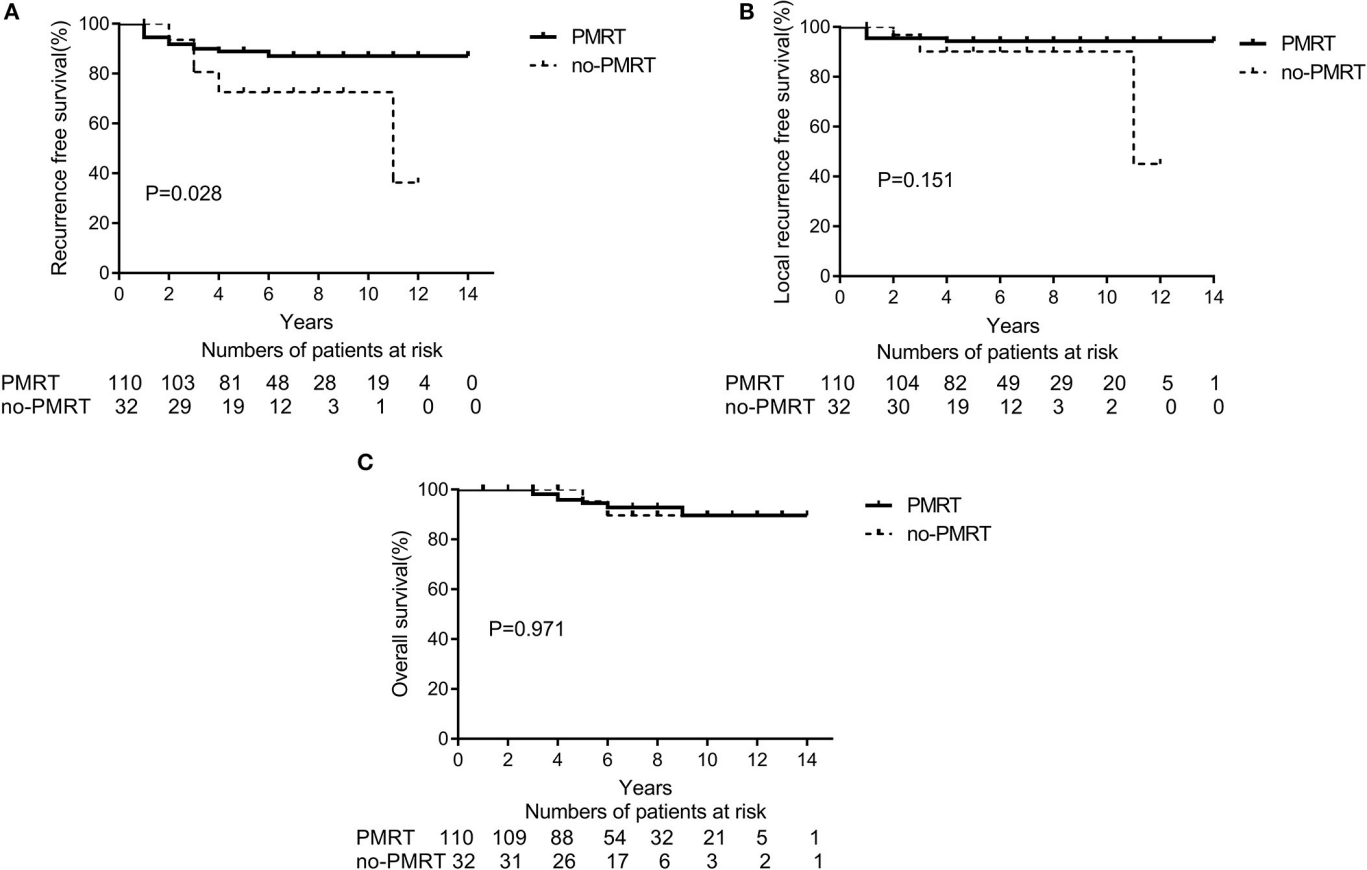
A Kaplan-Meier survival curve of non-matched groups according to post-mastectomy radiation therapy (PMRT) receipts. **(A)** Recurrence-free survival. **(B)** Local Recurrence-free survival. **(C)** Overall survival.

The 5-year RFS and OS rates for the entire population were 85.1 and 95.9%, respectively, and the 10-year RFS and OS rates were 83.5 and 89.2%, respectively. According to the univariate analyses, the 5-year RFS rates in the non-matched groups (PMRT group vs. non-PMRT group) were 88.7 and 72.4%, respectively (*p* = 0.028), and the 5-year LRFS rates in the PMRT and non-PMRT groups were 94.5 and 90.1%, respectively (*p* = 0.151). The corresponding 5-year OS rates were 96.1 and 95.0% (*p* = 0.971), respectively. The RFS, LRFS, and OS of PMRT are shown in Figures 1A–C. Clinical stage (2A vs. 2B) showed a significant trend with LRFS (*p* = 0.051). Age, response to NAC, HR status, HER-2 status, molecular subtype, LVSI, NAC regimen, and adjuvant chemotherapy, hormonal therapy, and targeted therapy did not affect the RFS, LRFS, and OS in the univariate analyses. According to multivariate analyses, PMRT was a significant prognostic factor affecting RFS (HR, 0.411; 95% CI, 0.175–0.968; *p* = 0.042, Table 3), but it did not affect LRFS or OS.

**Table 3:** Multivariate analyses of RFS before PSM and after PSM, Cox model (*n* = 142 and *n* = 96, respectively).

**Variables**	**Before PSM**			**After PSM**		
	**HR**	**95% CI**	***p*-value**	**HR**	**95% CI**	***p*-value**
Age group						
<50	0.563	0.229–1.383	0.210	0.360	0.112–1.162	0.087
≥50						
Clinical stage						
2A	0.556	0.232–1.332	0.188	0.388	0.140–1.075	0.069
2B						
Adjuvant radiotherapy						
Yes	0.411	0.175–0.968	0.042	0.323	0.115–0.913	0.033
No						

*RFS, recurrence-free survival; PSM, propensity-score matching; HR, hazards ratio; CI, confidence interval*.

### Univariate and Multi-Variate Analyses After PSM

**Figure 2:**
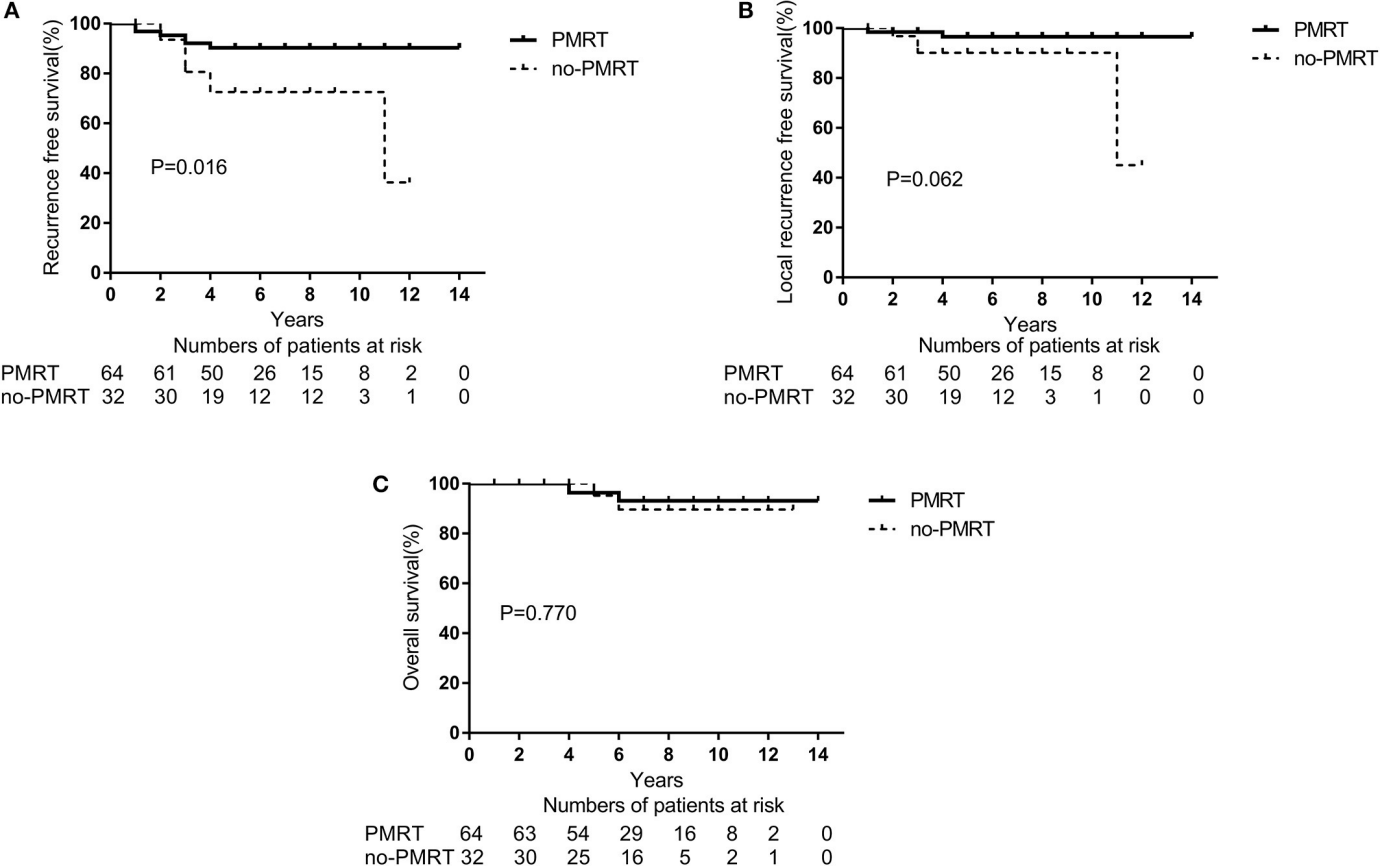
A Kaplan-Meier survival curve of groups after matching with PSM according to post-mastectomy radiation therapy (PMRT) receipts. **(A)** Recurrence-free survival. **(B)** Local Recurrence-free survival. **(C)** Overall survival.

With a PSM ratio of 1:2, a total of 96 patients (64 patients in the PMRT group and 32 patients in the non-PMRT group) were matched. In the univariate analyses, the 5-year RFS and LRFS rates were 90.1 and 96.9%, respectively, for the PMRT group, and 72.4 and 90.1% for the non-PMRT group (*p* = 0.016 and *p* = 0.062, respectively, Figures 2A,B). The corresponding 5-year OS rates were 96.7 and 95.0% (*p* = 0.770, Figure 2C). Clinical stage was significantly associated with RFS and LRFS (*p* = 0.047 and *p* = 0.013, respectively). Age, response to NAC, HR status, HER-2 status, molecular subtype, LVSI, NAC regimen, adjuvant chemotherapy, and adjuvant hormonal and targeted therapy did not affect the RFS, LRFS, or OS in the univariate analyses. In the multivariate analyses, the delivery of PMRT established a significant correlation with a difference in RFS (HR, 0.323; 95% CI, 0.115–0.913; *p* = 0.033, Table 3). The PMRT did not affect LRFS or OS.

### The Effect of PMRT in pCR Patients

**Figure 3:**
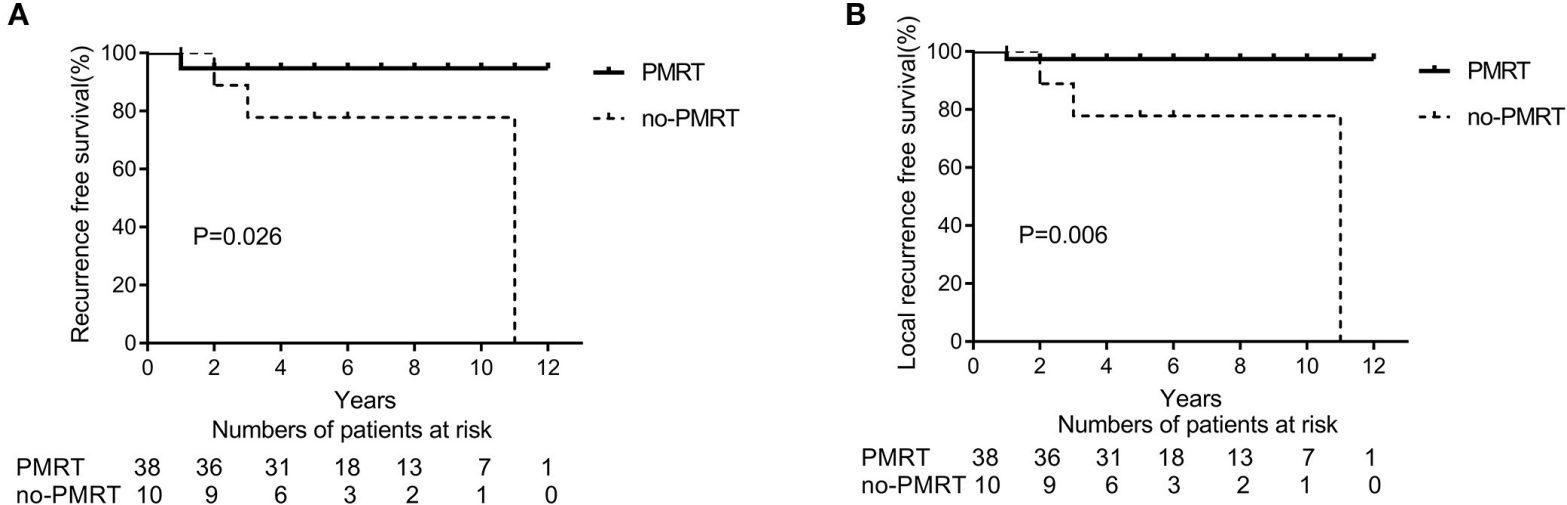
Recurrence-free survival (RFS) **(A)** and Local recurrence-free survival (LRFS) **(B)** in patients presenting with clinical stage II breast cancer and a complete pathological response (pCR) and treated with neoadjuvant chemotherapy and mastectomy with or without radiation therapy (PMRT group, *n* = 38 and non-PMRT group, *n* = 10).

After NAC, 48 (33.8%) patients had a pCR (ypT0 and ypN0) in the entire cohort. In the subtype of TNBC patients (*n* = 27), 11 (40.7%) patients achieved a pCR. In the subgroup analysis of 48 patients with pCR, the 5-year LRFS and DFS rates were significantly higher in the PMRT group (*n* = 38 patients) than in the non-PMRT group (*n* = 10 patients) (97.2 vs. 77.8%, *p* = 0.026, Figure 3A; 94.8 vs. 77.7%, *p* = 0.006, Figure 3B, respectively). Univariate analysis shows that PMRT is associated with higher LRFS and RFS rates; however, in multivariate analysis, clinical stage and PMRT did not predict any of these two outcomes (Table 4). None of the patients with pCR after NAC had died at the time of last follow up; as a result, the analysis of OS could not be conducted.

**Table 4:** Multivariate analyses of RFS in the pCR patients, Cox model (*n* = 48).

**Variables**	**HR**	**95%CI**	***p*-value**
Clinical stage			
2A	0.390	0.059–2.596	0.331
2B			
Adjuvant radiotherapy			
Yes	0.226	0.034–1.500	0.123
No			

*RFS, recurrence-free survival; pCR, complete pathological response; PSM, propensity-score matching; HR, hazards ratio; CI, confidence interval*.

## Discussion

NAC is used frequently in the treatment of clinical stage II breast cancer, raising issues regarding the indications for radiotherapy after mastectomy. NAC has been shown to modify the extent of pathological disease found in axillary lymph nodes in ~20–40% of patients (2, 3). The trastuzumab-based NAC regimen has shown a greater pCR rate (16, 17). A potential down-staging represents a challenge to the indications for PMRT. Whether clinically node-positive stage II patients with ypN0 after NAC would benefit from radiotherapy remains unclear, and evidence-based data are limited. In this study, we conducted an analysis of the largest cohort of stage II (T1-2N1) breast cancer patients with ypN0 after NAC and mastectomy reported to date in the literature. Our findings suggest that BC patients with stage II cancer who achieve ypN0 status after NAC and mastectomy might be suitable for PMRT.

Axillary lymph nodes can be down-staged with NAC; the potential for this change stresses the necessity of confirming the status of axillary nodes before NAC. The initial axillary node status of the patients with ypN0 after NAC included in previous studies was almost determined by image examination. However, axillary nodes have a certain false-positive rate. Reportedly, performing a biopsy of the sentinel lymph nodes prior to NAC treatment helps verify the axillary status of a patient (18); this avoids the interference of systemic therapy with axillary status and axillary dissection in sentinel node-negative patients and guides locoregional adjuvant treatment. In our study, the percentage of patients who underwent ultrasound-guided fine-needle aspiration or sentinel lymph node biopsy was 81.6%. However, only about half of the patients in the non-PMRT group had pathologic LN sampling. The problem that N-stage of some patients did not confirmed by biopsy indeed exist in the retrospective study and it was related to the determination of attending doctor and patient at the initial diagnosis. The clinical N stage of these patients was recorded as N1 (positive lymph nodes) according to the doctor's judgement. Under this circumstance, the axillary nodes of false-positive rates noted on imaging resulted that such patients might be upstaged. However, we speculated theoretically that the non-irradiation group may exist a small portion of patients with false-positive lymph nodes (N0) which may reduce the risk of locoregional recurrence. However, in such a condition, the RT-group had a better recurrence free survival (RFS) compared with non-irradiation group, which maybe indicated that radiation have a strength of improving the outcome. The current study confirmed the majority of patients with positive axillary nodes by pathological analysis and excluded patients with false-positive axillary lymph nodes as much as possible. This inclusion criterion makes our results more reliable than those of previous studies.

More patients received adjuvant chemotherapy in the non-PMRT group compared with patients in the PMRT group, because patients in the non-PMRT group may be at higher risk of recurrence due to not delivering PMRT, and adjuvant chemotherapy was an attempt to make up for this. After matching with PSM, there was no significant difference in the adjuvant chemotherapy.

After reviewing the existing literature, we found that a definitive conclusion regarding the effect of PMRT in the NAC setting is lacking (Table 5). The NCCN guidelines recommend that PMRT be based on the maximal disease stage at diagnosis and on post-chemotherapy pathology results. It is recommended that radiation be strongly considered for patients with clinical N1 and ypN0 (21). A National Surgical Adjuvant Breast and Bowel Project (NSABP) B51/RTOG 1304 trial is currently being conducted. The trial is investigating PMRT's role in breast cancer patients with clinical T1-3N1M0 who developed ypN0 after undergoing NAC treatment. The involved axillary node must be pathologically confirmed at diagnosis but SLNB is not permitted. The primary endpoint is invasive breast cancer recurrence–free interval. This important trial will address critical, previously unanswered questions in the future. However, most previous investigations of the role of PMRT in stage II BC patients with clinically positive nodes who developed ypN0 following NAC were retrospective analyses of studies with small sample size (8, 11). Le Scodan et al. (8) compared the outcomes of 39 stage II patients who received PMRT after developing ypN0 following NAC with the outcomes of 44 patients who did not receive PMRT and found no differences in the 5-year LRFS, DFS, or OS rates.

**Table 5:** Breast cancer patients with stage II who achieved ypN0 after neoadjuvant chemotherapy and mastectomy in past literature.

**References**	**Patients**	**Number of patients**	**PMRT vs. Non-PMRT**	**5-year LRR rate (%) (PMRT vs. Non-PMRT)**	**10-year LRR rate (%) (PMRT vs. Non-PMRT)**	**Median follow-up (Mo-)**
Huang et al. (19)	Stage I-II, ypN0 after NAC	32	12:20	–	0:0	69
McGuire et al. (10)	Stage II, pCR after NAC	30	20:10	–	0:0	62
Le Scodan et al. (8)	Stage II, ypN0 after NAC	83	39:44	2.6:7.1^*^	–	91.4
Shim et al. (11)	Stage II-III^§^, ypN0 after NAC	151	105:46	1.9:7.7^*^	–	57
Rong et al. (20)	Stage II, ypN0 after NAC	101	35:66	0:3.8^*^	–	70

*All studies enrolled in this table were retrospective. NAC, neoadjuvant chemotherapy; PMRT, post-mastectomy radiotherapy; LRR, locoregional recurrence*.

§ *This study did not separate stage II patients from their stage III counterparts*.

* *p > 0.05*.

Similarly, a multicenter retrospective study of a Korean population (*n* = 151) (11) also demonstrated that clinical stage II–III breast cancer patients with pN0 following NAC did not benefit from PMRT. According to these studies, there was a favorable outcome, and stage II patients who developed ypN0 following treatment with NAC and mastectomy had a lower risk of locoregional relapse. Our study revealed that the prognosis of patients with ypN0 statuses after NAC was favorable, consistent with the findings of previous studies. We also established an association between PMRT and better RFS in BC patients who had initial stage II of the disease and ypN0 after NAC treatment. The negative results obtained in previous studies could have resulted from the limited numbers of patients included in those studies or from a low total number of events and considerable variations in the factors of prognosis (e.g., clinical T or N during diagnosis) between PMRT-receiving and non-PMRT-receiving patients. The present study includes the largest number of stage II patients with ypN0 after NAC analyzed to date, and the PSM analysis balanced the difference(s) in potentially important prognostic factors between the PMRT and non-PMRT groups. Patients with ypN0 and residual disease represent an intermediate-risk group. The analysis conducted in this study indicates that the tailoring of PMRT might place these patients at increased risk of recurrence.

NAC modifies the pathological extent of a disease. The newer NAC regimens have increased the percentage of pCR (16, 17, 22). One study reported that 46.8% of HER2-positive breast cancer patients subjected to neoadjuvant chemotherapy with trastuzumab achieved a pCR (16). However, few published studies have demonstrated how pCR status affects locoregional treatment choices. The indications for PMRT following pCR to NAC remain unclear, especially in the early stage of the disease. Findings from a retrospective study (10) performed on BC patients who achieved pCR after NAC suggest that post-mastectomy radiation therapy is potentially not a necessity for certain patients, such as stage II patients [PMRT-receiving patients (*n* = 10) and non-PMRT-receiving patients (*n* = 20); both had 10-year LRR rates of 0%]. Huang et al. also reported that there were no differences in LRR rates for clinical stage I or II patients who attained pCR status, and none of the 32 patients tested developed LRR [RT-receiving patients (n = 12) and non-RT-receiving patients (n = 20); both had 10-year LRR rates of 0%] (19). The results of our study indicate that PMRT did not correlate with RFS in stage II BC patients with pCR after NAC and mastectomy. Although the sample size of this population was limited, it remains the largest cohort of pCR patients studied to date. A preliminary viewpoint inferred from this analysis is that PMRT may be omitted for these pCR patients.

The present study has some limitations. Given the imbalance in sample size between the PMRT and non-PMRT groups, it is imperative that care be taken in interpreting the data. Selection bias, such as the inherent shortcomings of a retrospective study, may affect the differences in outcomes in the PMRT and non-PMRT groups. In some of the patients in the cohort, initial axillary node status was not determined by pathological analysis. The clinical-stage is not equal to the pathological stage. The non-irradiation group may include a small number of patients with false positive lymph nodes (N0), reducing the incidence of locoregional recurrence.

In conclusion, a decrease in recurrence-free survival was observed when PMRT was omitted in stage II BC patients with ypN0 after NAC and mastectomy in this study. PMRT might, therefore, be necessary for stage II (T1-2N1M0) patients with ypN0 after NAC and mastectomy. However, the benefit of PMRT for these patients requires assessment in further prospective studies.

## Data Availability Statement

The raw data supporting the conclusions of this article will be made available by the authors, without undue reservation.

## Ethics Statement

This study was reviewed and approved by the Institutional Review Board of Chinese PLA General Hospital. Because of the retrospective nature of the study, patient consent for inclusion was waived.

## Author Contributions

BS and WZ: conceptualization. QW, LZ, and XH: methodology and validation. QW, JZha, PE, XM, JS, HS, JZhu, and LZ: formal analysis. QW and JZha: investigation. LZ, WZ, and BS: data curation. QW and JZha: writing—original draft preparation. XH, PE, XM, SW, WZ, and BS: writing—review and editing. All authors have approved the final manuscript.

## Conflict of Interest

The authors declare that the research was conducted in the absence of any commercial or financial relationships that could be construed as a potential conflict of interest.
